# An adaptive landscape for training in the essentials of next gen sequencing data acquisition and bioinformatic analysis

**DOI:** 10.1186/1471-2105-15-S10-P25

**Published:** 2014-09-29

**Authors:** Mark Farman, Patrick Calie, Jerzy Jaromczyk, Jolanta Jaromczyk, Neil Moore, Daniel Harris, Chris Schardl

**Affiliations:** 1 Department of Plant Pathology, University of Kentucky, Lexington, KY 40546, USA; 2 Advanced Genetic Technologies Center, University of Kentucky, Lexington, KY 40456, USA; 3 Department of Biological Sciences, Eastern Kentucky University, Richmond, KY 40475, USA; 4 Department of Computer Science, University of Kentucky, Lexington, KY 40546, USA

## Background

Recent technological advances in Next Generation Sequencing (NGS) have reduced both the cost and time required to produce Large Data Sets (LDS) of nucleotide sequences. These advances have led to an exponential proliferation of nucleotide sequence data coupled with an exacerbation of a persistent conundrum: the level of difficulty in generating LDS is rapidly decreasing, but the exposure, development and training of students and investigators in the bioinformatic approaches requisite to the proper and correct analysis of such data sets is experiencing a parallel increase in difficulty.

## Materials and methods

To address this national need, we have developed a client-oriented Summer Workshop in NGS technology and related bioinformatics that combines a practical experience to the biological and technical aspects of NGS coupled with an integrated bioinformatic exposure to a suite of appropriate analytical tools and approaches. The biological component utilizes experiences in both semi-conductor and sequencing by synthesis technologies with nucleic acid samples provided to the Workshop clients. This allows clients to understand issues of DNA quality, biological sample contamination, and preliminary quality assessment of the output data. The bioinformatic modules then use the client-generated data as working platforms to allow the clients to engage in such activities as assessing sequence quality, *de novo* genome assembly, alignment of RNASeq data to a reference genome, construction of *in silico* gene models, and populating a genome browser, to name a few. The global perspective of the workshop is to develop client skills in formulating appropriate scientific questions prior to investing in NGS approaches, in choosing the most appropriate NGS platform (pyrosequencing, semi-conductor sequencing, or sequencing by synthesis) suitable for their specific questions, and in the efficient and correct analysis of genomic and transcriptomic data sets. In this, our third year, we continue to modify and adapt the Workshop curriculum to address the evolving needs of our clients, based upon our experiences with real time data from our Advanced Genetic Technologies Center at the University of Kentucky. One anticipated outcome is the future development of a virtual community of former and current Workshop participants who can share experiences and insights as NGS technologies and allied bioinformatic approaches evolve through time.

